# Draft Genome Sequence of Enterobacter asburiae NCR1, a Plant Growth-Promoting Rhizobacterium Isolated from a Cadmium-Contaminated Environment

**DOI:** 10.1128/MRA.00478-21

**Published:** 2021-09-02

**Authors:** Charlotte J. François, Steven Batinovic, Steve Petrovski, Anthony R. Gendall

**Affiliations:** a Department of Animal, Plant and Soil Sciences, La Trobe University, Bundoora, Victoria, Australia; b Department of Physiology, Anatomy and Microbiology, La Trobe University, Bundoora, Victoria, Australia; University of Maryland School of Medicine

## Abstract

Enterobacter asburiae NCR1 is a plant growth-promoting rhizobacterium isolated from the rhizosphere of *Carpobrotus rossii*. We report the draft genome sequence of *E. asburiae* strain NCR1, which revealed many genes facilitating beneficial interactions with plant hosts.

## ANNOUNCEMENT

The genus Enterobacter comprises more than 20 species that are widely distributed through various environments. Some species of Enterobacter can enhance plant growth by increasing the availability of nutrients and water and synthesizing plant growth-promoting hormones (1, 2). Enterobacter asburiae NCR1 was previously isolated from the rhizosphere of Carpobrotus rossii grown in a cadmium-contaminated environment (3).

*E. asburiae* NCR1 was grown at 27°C for 16 h in Luria-Bertani broth, and total genomic DNA was extracted using the Bioline Isolate II genomic DNA kit and converted to sequencing libraries using the Nextera XT DNA library prep kit (Illumina) with 1 ng of input DNA, according to the manufacturer’s instructions. The quality and concentration of the genomic DNA were assessed using an Invitrogen Qubit 3.0 fluorometer. The libraries were sequenced on an Illumina MiSeq instrument using a MiSeq v.3 reagent kit (600 cycles), which generated 919,623 reads of 300-bp paired-end reads. The sequence reads were trimmed to a Phred threshold quality score of 20 using Trimmomatic v.0.36.6 (4), and assembly was performed using SPAdes v.3.12.0 (5), both on Galaxy Australia (6). The total length of the genome sequence of *E. asburiae* NCR1 was 4,546,475 bp, with a G+C content of 56.08%. The final number of contigs was 15, with an *N*_50_ value of 2,597,842 bp, and the final genome coverage was 50×. Default parameters were used unless otherwise stated.

The annotation of the assembled genomic sequence was performed using the Prokaryotic Genome Annotation Pipeline (PGAP) v.4.10 of the National Center for Biotechnology Information (NCBI) (7). The genome sequence of *E. asburiae* NCR1 was closely related to that of *E. asburiae* GN04222 with an average nucleotide identity (ANI) of 98.06%, as calculated by a JSpeciesWS v.3.8.2 analysis (8). A total of 4,237 genes were identified, with 4,240 coding sequences (CDS), including 87 RNA genes. The RNA genes comprised 4 5S rRNAs, 1 partial 16S rRNA, 1 partial 23S rRNA, and 73 tRNA genes.

The annotated genome sequence revealed several genes pertinent to plant-microbe interactions, including those involved in auxin synthesis (*iaa* and *ipdC*) and phosphorus metabolism (*phoP*). Bacterially derived auxin can enhance the growth of plants, while the catabolism of phosphates may improve phosphate availability to the host plants (9).

Heavy metal resistance to Zn, Cd, and Pb is conferred through *zntA*, which acts as a P-type ATPase pump (10) and is suggestive of this strain’s ability to survive in challenging environments. This draft genome sequence of *E. asburiae* NCR1 could provide valuable information regarding the plant growth-promotive properties and heavy metal resistance and tolerance of this strain.

### Data availability.

The *E. asburiae* NCR1 whole-genome sequencing project has been deposited at GenBank under accession number JAAAJX000000000, BioProject accession number PRJNA600757, and BioSample accession number SAMN13831070. The raw sequences were deposited in the Sequence Read Archive under accession number SRR14460571.

## References

[1] Ramesh A, Sharma SK, Sharma MP, Yadav N, Joshi OP. 2014. Plant growth-promoting traits in *Enterobacter cloacae* subsp. *dissolvens* MDSR9 isolated from soybean rhizosphere and its impact on growth and nutrition of soybean and wheat upon inoculation. Agric Res 3:53–66. doi:10.1007/s40003-014-0100-3.

[2] Jha CK, Aeron A, Patel BV, Maheshwari DK, Saraf M. 2011. Enterobacter: role in plant growth promotion, p 159–182. In Maheshwari DK (ed), Bacteria in agrobiology: plant growth responses. Springer, Berlin, Germany. doi:10.1007/978-3-642-20332-9_8.

[3] Liu W, Wang Q, Wang B, Hou J, Luo Y, Tang C, Franks AE. 2015. Plant growth-promoting rhizobacteria enhance the growth and Cd uptake of *Sedum plumbizincicola* in a Cd-contaminated soil. J Soils Sediments 15:1191–1199. doi:10.1007/s11368-015-1067-9.

[4] Bolger AM, Lohse M, Usadel B. 2014. Trimmomatic: a flexible trimmer for Illumina sequence data. Bioinformatics 30:2114–2120. doi:10.1093/bioinformatics/btu170. 24695404 PMC4103590

[5] Bankevich A, Nurk S, Antipov D, Gurevich AA, Dvorkin M, Kulikov AS, Lesin VM, Nikolenko SI, Pham S, Prjibelski AD, Pyshkin AV, Sirotkin AV, Vyahhi N, Tesler G, Alekseyev MA, Pevzner PA. 2012. SPAdes: a new genome assembly algorithm and its applications to single-cell sequencing. J Comput Biol 19:455–477. doi:10.1089/cmb.2012.0021. 22506599 PMC3342519

[6] Afgan E, Sloggett C, Goonasekera N, Makunin I, Benson D, Crowe M, Gladman S, Kowsar Y, Pheasant M, Horst R, Lonie A. 2015. Genomics virtual laboratory: a practical bioinformatics workbench for the cloud. PLoS One 10:e0140829. doi:10.1371/journal.pone.0140829. 26501966 PMC4621043

[7] Tatusova T, DiCuccio M, Badretdin A, Chetvernin V, Nawrocki EP, Zaslavsky L, Lomsadze A, Pruitt KD, Borodovsky M, Ostell J. 2016. NCBI Prokaryotic Genome Annotation Pipeline. Nucleic Acids Res 44:6614–6624. doi:10.1093/nar/gkw569. 27342282 PMC5001611

[8] Richter M, Rosselló-Móra R, Oliver Glöckner F, Peplies J. 2016. JSpeciesWS: a Web server for prokaryotic species circumscription based on pairwise genome comparison. Bioinformatics 32:929–931. doi:10.1093/bioinformatics/btv681. 26576653 PMC5939971

[9] Spaepen S, Vanderleyden J. 2011. Auxin and plant-microbe interactions. Cold Spring Harb Perspect Biol 3:a001438. doi:10.1101/cshperspect.a001438. 21084388 PMC3062209

[10] Naz N, Young HK, Ahmed N, Gadd GM. 2005. Cadmium accumulation and DNA homology with metal resistance genes in sulfate-reducing bacteria. Appl Environ Microbiol 71:4610–4618. doi:10.1128/AEM.71.8.4610-4618.2005. 16085855 PMC1183370

